# Does Simple “Reassurance” Work in Patients with Medically Unexplained Physical Symptoms?

**DOI:** 10.1371/journal.pmed.0030313

**Published:** 2006-08-01

**Authors:** Javier I Escobar

## Abstract

Escobar discusses a new study examining the impact of reassurance in three groups: patients with medically unexplained physical symptoms compared, healthy controls, and patients with depression.

The humanistic principles of the medical profession are nicely condensed in Oliver Wendell Holmes's old maxim stating that the key role of the physician is “to comfort always.” Thus, beyond the specificity of diagnoses and the effectiveness of treatments, the traditional practice of medicine gave high importance to bedside manner and interpersonal issues.

In contrast, 21st century medicine, with its reliance on technology and the changes in practice patterns (e.g., managed care), leaves little time for face-to-face interactions, gradually eroding the doctor–patient relationship. In this transition towards technological medicine, it would appear that we have switched from “comforting” our patients (where “to comfort” means “to give strength and hope; to ease the grief or trouble; to cheer” [1]) to simply “reassuring” them (where “to reassure” means “to assure anew; or restore to confidence” [1]). In this modern context, “reassurance” simply means to let patients know that their symptoms do not appear to be caused by physical disease.

In primary care, the de facto mental health system [2], large numbers of patients with common mental disorders such as depression or anxiety present with medically unexplained physical symptoms (MUPS). These somatic presentations have been well documented throughout the years, have always baffled the medical establishment, and metamorphose with cultural evolution and the changing perspectives of medical paradigms [3]. In all their forms, patients presenting with MUPS are the bane of modern medicine. Not fitting anywhere, they land in the vortex of the centuries-old mind–body debate.

In primary and specialty care nowadays, a number of “functional labels” [4] are a convenient though imprecise way to frame certain types of MUPS. These functional labels seem more acceptable to patients than receiving no diagnosis or having their symptoms attributed to a mental disorder, often viewed as a moral infirmity. MUPS add complexity to other physical or psychiatric disorders and lead to additional impairment and complications, so it is imperative that these presenting symptoms be properly addressed.

Patients presenting with medically unexplained physical symptoms are the bane of modern medicine.

## A New Study on MUPS

In a study by Rief et al. published in *PLoS Medicine*, the authors examined the impact of reassurance in three separate groups: 30 healthy controls, 22 depressed patients without MUPS, and 33 patients with MUPS [5]. All patients listened to a short audiotaped medical report about a person with abdominal pain visiting a doctor to receive test results. The report included ten items discussing possible explanations for abdominal pain (such as stomach ulcer and bowel cancer), with reassurance that all these conditions were either totally ruled out (e.g., “The reason for your complaints is definitely not a stomach flu”) or very unlikely (e.g., “With this finding we don't believe that you have bowel cancer; this is very unlikely”). In other words, in the end there was no medical explanation for the symptoms. The research participants also listened to two control reports—a report of a social situation (a person learns from a friend that he/she is not invited to a party) and a report of a neutral situation (a person with car problems who is told by a mechanic the possible reasons why the car is not working).

In this study, the clinical reality of today's busy primary care clinic was reflected in the design. Thus, when “reassuring,” the physician simply uttered brief, mechanical statements such as “we do not believe you have cancer” or “you do not have an ulcer” to convey the fact that there were no positive findings in the physical examination and laboratory studies. The results of the study showed that of the three groups of patients, those presenting with MUPS were less likely than patients in the other two groups to accurately remember the fact that the physician emphasized lack of medical explanation for the symptoms.

For each of the ten items in the audiotaped medical report, all participants were asked to rate the likelihood (on a visual analog scale from zero, absolutely unlikely, to 100, absolutely likely) of a medical explanation for the symptoms discussed in the audiotape. For example, after hearing in the report “You also don't have a stomach ulcer; I definitely would have seen it in the ultrasound examination,” the participants were asked “What does your doctor think the likelihood is you have stomach ulcer?” The mean estimate by the patients with MUPS for the likelihood of a medical explanation was 15%, compared with 10% for the patients with depression and 5% for the healthy controls. These differences reached statistical significance.

The authors' interpretation of their findings is that there is as a failure of memory that may be intrinsic to patients with MUPS. However, although the differences between the three groups were statistically significant, patients with MUPS assigned a 15% likelihood to medical causes (scale of 0–100), which is still a small likelihood, and therefore a question arises about the practical significance of these findings. An alternative explanation for the finding of a different response by patients with MUPS is that their previous experiences in dealing with the medical establishment may have influenced their perceptions.

## Limitations of the Study

A major omission in this design was that the authors did not include a group of patients with bonafide medical disorders and therefore one cannot say confidently that the trait of assigning a medical explanation for unexplained physical symptoms applies only to patients with MUPS. Such a comparison group of service users would have also allowed the researchers to examine whether this trait was due to a memory problem or whether it reflected previous experiences in medical care. Excluding depressed patients with somatic symptoms may result in an atypical group given the fact that most patients with depression may present to physicians with only physical symptoms [6,7] and that most primary care patients with MUPS have a psychiatric disorder such as depression [8].

## Implications for Clinical Practice

Findings from this study support previous anecdotal observations indicating that simple “reassurance” does not work well in patients with MUPS. To date, the best studied intervention for MUPS has been a psychiatric consultation letter [9]. This consists of a brief letter sent to primary care physicians recommending that they examine patients with MUPS during regularly scheduled appointments, perform brief physical examinations focusing on the area of discomfort at each visit, avoid unnecessary diagnostic procedures, invasive treatments, and hospitalizations, avoid using statements such as “symptoms are all in your head,” and briefly allow and encourage patients to talk about “stressors.” This approach may be a more effective framing of “reassurance” than the mechanical statements described above. The ideal model, I believe, would be to incorporate expert mental health consultation into primary care routines.

## Abbreviations

MUPS: medically unexplained physical symptoms
